# Intraventricular hemorrhage in neonates born before 32 weeks of gestation—retrospective analysis of risk factors

**DOI:** 10.1007/s00381-016-3127-x

**Published:** 2016-05-28

**Authors:** Dawid Szpecht, Marta Szymankiewicz, Irmina Nowak, Janusz Gadzinowski

**Affiliations:** 1 Chair and Department of Neonatology, Poznan University of Medical Sciences, Polna 33 Street, 60-535 Poznań, Poland; 2 Poznan University of Medical Sciences, Poznań, Poland

**Keywords:** Intraventricular hemorrhage, Preterm newborns, Risk factors

## Abstract

**Introduction:**

Intraventricular hemorrhage (IVH) affects 15–20 % of babies born before 32 weeks of pregnancy. A lot of risk factors of developing IVH are known. The making appropriate recommendations for dealing with infant born less than 32 weeks of gestation aimed at reducing the incidence of IVH is still needed. The study aim was to determine the incidence and analyze risk factors of IVH stage 3 and 4 in infants born before 32 + 0 weeks of pregnancy.

**Methods:**

The retrospective analysis of 267 preterm babies (24 to 32 weeks of gestation) hospitalized in 2011–2013 at Department of Neonatology, Poznan University of Medical Sciences was performed. The diagnosis of IVH was confirmed by ultrasound scans according to Papille criteria. Stage 3 and 4 of IVH was confirmed in 14 (25 %) newborns from 23 to 24 weeks of gestation; 21 (37.5 %) from 25 to 26 weeks of gestation; 11 (19.6 %) from 27 to 28 weeks of gestation; 9 (16.1 %) from 29 to 30 weeks of gestation; and 1 (1.8 %) from 31 to 32 weeks of gestation.

**Result:**

The incidence of IVH stage 3 and 4 was higher in children: with less use of AST (OR 1.27; 0.62–2.61), born out of third-level hospitals (OR 2.25; 1.23–4.08), born with asphyxia (OR 3.46; 1.8–6.64), with acidosis treated with NaHCO3 (OR 6.67; 3.78–11.75), those who in the first days of life were treated for hypotension (OR 9.92; 5.12–19.21).

**Conclusion:**

No or uncompleted antenatal steroid therapy increased probability for development of severe intraventricular hemorrhage. Antenatal steroids therapy should be promoted among women at risk of a premature delivery. Hypotension therapy with catecholamines and acidosis with sodium hydrogen carbonate should be carefully considered. The use of appropriate prophylaxis of perinatal (antenatal steroids therapy women at risk of preterm birth, limiting the indications for the use of catecholamines for hypotension treatment and sodium hydrogen carbonate for acidosis therapy, limitation of preterm deliveries outside tertiary referral centeres) significantly reduces the incidence of intraventricular hemorrhage stage 3 and 4. The significance of intraventricular hemorrhage creates a need to carry out periodical analysis, at regional level, concerning its incidence, causes and effects to improve local treatment outcomes by identifying further courses of action.

## Introduction

Intraventricular hemorrhage (IVH), characterized as bleeding due to rupture of blood vessels within the germinal matrix tissue of the developing brain into the ventricular system and the incidence for IVH grades I–IV, is around 27 % in neonates weighing less than 1500 g [1]. IVH ranges in severity from grade I to the most severe grade IV. About 90 % cases of intraventricular hemorrhage occur within the first 3 days of the newborn’s life and in 20–40 % of IVH cases become more extended, during first week of life. In the majority of cases involving mild bleeding (classified as grade 1 and grade 2), no clinical effects are observed, usually resolve themselves and cause no long-term problems. Approximately in 60 % of premature infants with grade III and IV of IVH incur cognitive disabilities such as cerebral palsy and mental retardation [2].

## Material and methods

### Study population

Two hundred sixty-seven premature infants (delivery before 32 + 0 weeks of gestation) admitted to the Neonatal Intensive Care Unit at the Department of Neonatology, Poznań University of Medical Sciences between 2011 and 2013 were recruited into study.

The study will not include neonates born after 32 + 0 weeks of pregnancy, as well as those born from multiple pregnancies, from pregnancies complicated by death of one of the fetuses, chromosomal abnormalities or TORCH (toxoplasmosis, other, rubella, cytomegalovirus, herpes).

Demographics of this population are described in Table 1.

**Table 1:** Characteristic of study population

	Group without IVH and with IVH I and II stage (*N* = 211; 79 %)	Group with IVH stage III and IV (*N* = 56; 21 %)	*p*
Gender			0.853^a^
Male	116 (55 %)	28 (50 %)	
Female	95 (45 %)	28 (50 %)	
Gestational age (week)			0.019^a^
23–24	24 (11.4 %)	14 (25 %)	
25–26	65 (30.8 %)	21 (37.5 %)	
27–28	54 (25.6 %)	11 (19.6 %)	
29–30	45 (21.3 %)	9 (16.1 %)	
31	23 (10.9 %)	1 (1.8 %)	
Birth weight (gram)			0.464^a^
<750	38 (18 %)	14 (25 %)	
750–1000	70 (33.2 %)	21 (37.5 %)	
1000–1500	71 (33.6 %)	15 (26.8 %)	
>1500	32 (15.2 %)	6 (10.7 %)	
SGA			0.747^b^
Yes	7 (3.3 %)	2 (3.6 %)	
No	204 (96.7 %)	54 (96.4 %)	
AST			0.014^a^
Yes	167 (79.1 %)	35 (62.5 %)	
No or uncompleted	44 (20.9 %)	21 (37.5 %)	
Mode of delivery			0.277^a^
Vaginal	86 (40.8 %)	28 (50 %)	
Cesarean section	125 (59.2 %)	28 (50 %)	
Third level hospitals			0.005^a^
Inborn	181 (85.8 %)	42 (75 %)	
Outborn	30 (14.2 %)	14 (25 %)	
Asphyxia			0.052^a^
Yes	29 (13.7 %)	13 (23.2 %)	
No	162 (86.3 %)	43 (76.8 %)	
Intrauterine infection			0.880^a^
Yes	94 (44.5 %)	26 (46.4 %)	
No	117 (55.5 %)	30 (53.6 %)	
Hypotension therapy			0.002^a^
Yes	131 (62.1 %)	47 (83.9 %)	
No	80 (37.9 %)	9 (16.1 %)	
Acidosis therapy			0.003^a^
Yes	122 (57.8 %)	45 (80.4 %)	
No	89 (42.2 %)	11 (19.6 %)	
Blood coagulation disorders and/or thrombocytopenia			0.807^b^
Yes	4 (1.9 %)	2 (3.6 %)	
No	207 (98.1 %)	54 (96.4 %)	

^a^Chi-square test

^b^Chi-square test with Yate’s correction

### Diagnosis of intraventricular bleeding

Intraventricular hemorrhage was diagnosed with the use of a cranial ultrasonographic scan. According to the guidelines of the American Academy of Neurology (AAN), a routine cranial ultrasonographic scans were performed on 3rd, 7th day of life, and once more just before discharging from hospital. The classification of intraventricular bleeding was based on the Papille IVH classification [3]. In study group diagnosed 110 patients with IVH stage 1; 101 patients with IVH stage 2; 49 patients with stage 3; and 7 patients with stage 4.

### Risk factors

We explored the relationship between the occurrence of IVH and the following prenatal and perinatal variables: gender, gestational age (GA; weeks), birth weight (BW, grams); antenatal steroids therapy (AST, betamethasone 12 mg intramuscularly every 24 h for two doses); small for gestational age (SGA, defined as birth weight under 3rd percentile); type of delivery (vaginal birth vs. cesarean section); delivery outside third-level hospitals, birth asphyxia (defined as APGAR score less than 6 at 10 min and ph < 7.0 or blood base excess (BE) < −15 mmol/l in cord blood); intrauterine infection (defined as positive culture in sterile originally accompanied by clinical symptoms); therapy in first 7 days of life with crystalloids (bolus 10–15 ml/kg) and/or catecholamines of hypotension (defined as mean blood pressure below value corresponding to neonate’s gestational age), treatment of the acidosis with NaHCO3 (when blood pH was below 7.2 and/or BE less than −10 mmol/l); blood coagulation disorders (defined as prolonged the prothrombin time (PT) below 65 % and/or increased International Normal Ratio (INR) more than 1.5 and/or prolonged activated partial thromboplastin time (APTT) more than 45 s; thrombocytopenia (defined as platelet count less than 100,000 per microliter of blood) in neonate on developing IVH.

### Statistical analysis

Chi-square test without or with Yates correction were applied for comparisons of dichotomous variables, where appropriate. The odds ratio (OR) and 95 % confidence intervals (95 % CI) were calculated. Unconditional logistic regression analysis was used to adjust for the effect of confounders such as gender, GA, BW; AST, SGA, outborn patients, birth asphyxia, intrauterine infection; hypotension, acidosis, blood coagulation disorders, thrombocytopenia was used to make adjustment of parameters. A *P* value below 0.05 was judged to be statistically significant.

Aforementioned statistical calculations were performed using CytelStudio version 10.0, created January 16, 2013 (CytelStudio Software Corporation, Cambridge, USA), and Statistica version 10, 2011 (Stat Soft, Inc., Tulsa, USA).

## Results

The incidence of IVH stage 3 and 4 was comparable in female (*n* = 28; 50 %) and male (*n* = 28; 50 %) neonates with no significance. The risk of IVH stage 3 and 4 was the greater the lower the gestational age and was significantly higher in children born 23–24 weeks of gestation (OR 18.39; 6.4–54.98), 25–26 weeks of gestation (OR 9.63; 3.98–25.48), 27–28 weeks of gestation (OR 5.52; 2.1–15.53) and 29–31 weeks of gestation (OR 3.82; 1.31–11.51) comparing to children born after 32 weeks of gestation. The incidence of IVH stage 3 and 4 was higher in children: with less use of AST (*p* = 0.014; OR 1.27; 0.62–2.61), born out of third-level hospitals (*p* = 0.007; OR 2.25; 1.23–4.08), born with asphyxia (*p* < 0.0001; OR 3.46; 1.8–6.64), with acidosis treated with NaHCO3 (*p* < 0.0001; OR 6.67; 3.78–11.75), those who in the first days of life were treated for hypotension (*p* < 0.0001; OR 9.92; 5.12–19.21). There was no significance increase incidence of IVH stage 3 and 4 in neonates spontaneous delivered vs by cesarean section, children with SGA vs AGA, children with intrauterine infection and neonates with blood coagulation disorders and/or thrombocytopenia after birth.

Multivariate analysis revealed that the more risk factors present the greater chance incidence of IVH 3 and 4 stage and in children born before 32 weeks of gestation and without AST (*p* = 0.0387; OR 1.27; 0.62–2.61), and born with asphyxia (*p* = 0.0029; OR 1.52; 0.67–3.46), and hypotension (*p* < 0.0001; OR 4.35; 1.81–10.47) and with acidosis (*p* < 0.0001; OR 5.17; 2.22–12.03).

Seven of 267 (2.6 %) patient died, including 2 of 38 (5.3 %) newborns from 23 to 24 weeks of gestation; 2 of 86 (2.3 %) from 25 to 26 weeks of gestation; 1 of 65 (1.5 %) from 27 to 28 weeks of gestation; 2 of 54 (3.7 %) from 29 to 30 weeks of gestation and none from 31 weeks of gestation. Five of 56 (8.9 %) patients with stage III and IV of IVH died, of which none was born respectively in 23–24; 2 (9.5 %) in 25–26; 1 (9 %) in 27–28; 2 (22.2 %) in 29–30 and none in 31 weeks of gestation.

## Discussion

The role of hypotension and its involvement in the pathogenesis of complications in preterm infants is not clear. Research into various factors involved in the etiopathogenesis of IVH list hypotension among the risk factors for it. Different results were presented by Batton et al. [4] who did not find significant differences in the psychomotor development between newborn infants who were treated for hypotension and those who were not. Similarly, Alderliesten et al. [5] did not determine an association between hypotension and the development of adverse neurological outcomes. Therefore, a question may be asked whether hypotension among newborn infants should be treated or not. Current guidelines in many countries (e.g., USA, the UK, Poland) are similar. The first line of therapy is crystalloid fluid infusion to eliminate hypovolemia as the potential reason for hypotension. The next line of therapy is catecholamines, introduced in the following order: dopamine at 10–20 μg/kg/min, optionally combined with dobutamine at 20–40 μg/kg/min. If there is no therapeutic effect after dopamine and dobutamine have been administered, adrenalin or noradrenalin is recommended, at respective doses of 0.05 and 0.1–1 μg/kg/min. The next step is the use of corticosteroids: 1–2 mg of hydrocortisone per kg of body weight given every 6–8 h [6]. Unfortunately, as demonstrated in our study, catecholamine therapy increased the risk of development of IVH (grade 3 and 4) among infants born before week 32 of gestational age. The results of other research are comparable. In their study carried out on a group of 153 neonates, Rong et al. [7] found catecholamine therapy to be a risk factor for IVH. An American study of infants with VLBW confirmed an increased risk of IVH development and higher mortality rates among the neonates treated for hypotension. Moreover, in this group, a higher risk of later complications, including hearing impairment or cerebral palsy, was observed, compared to infants without hypotension or hypotensive untreated infants. This might suggest that refraining from treatment of hypotension is safer for premature infants than catecholamine therapy. Alternative forms of treatment should also be considered. Ibrahim et al. [8] found that hydrocortisone used to treat hypotension had similar effectiveness to dopamine. Moreover, when compared to placebo, hydrocortisone does not increase the risk of IVH nor infant mortality rates. The appropriate criteria of initiating catecholamine treatment are not established. One of the most popular criteria is a fall of blood pressure 5 mmHG less than correct level and the presence of at least two indicators of hypoperfusion, such as average blood pressure lowered by 3 mmHG, lactate >4 mmol/l, capillary refill time >4 s [9]. In their other research, Batton et al. initiated hypotension treatment on 203 neonates born between 23rd and 26th week of pregnancy, basing on 15 different definitions of hypotension. In all treated groups, higher risk of appearing IVH and lower survival rate were observed. Independently from the moment of administration, catecholamines caused higher risk of appearance of the aforementioned complications [10]. Currently in preterm infants should be taken into consideration the permissive hypotension. A numerical blood pressure value lower than gestational age should not be used as the only indicator for treating early period hypotension in VLBW. Hypotension treatment should be administered based on clinical condition of patients [11].

Another risk factor for grade 3 and 4 IVH, confirmed also in our study, is a lack of AST in pregnant women at risk of a premature delivery. The main objective of the use of steroid therapy is to prevent respiratory distress syndrome (RDS) by stimulating maturity of fetal respiratory system. AST stimulate development of type 1 and type 2 pneumocytes. Induction of type 2 pneumocytes increases surfactant production by inducing production of surfactant proteins and enzymes necessary for phospholipid synthesis. Moreover, AST accelerate absorption of lung fluid after the birth due to induce of pulmonary beta-receptors, fetal lung antioxidant enzymes, and upregulation of gene expression for the epithelial Na+ channel [12]. A Cochrane review showed that AST reduced the risk of neonatal death by 31 %, RDS by 44 %, and IVH by 46 % [13]. A 2016 meta-analysis of randomized trials of antenatal corticosteroid therapy before 24 weeks of gestation demonstrated a reduction in perinatal mortality at 23 weeks of gestation (OR 0.45, 95 % CI 0.33–0.60), and a possible reduction at 22 weeks (OR 0.66, 95 % CI 0.40–1.07). No statistical reductions were observed for respiratory distress syndrome, severe intraventricular hemorrhage, or necrotizing enterocolitis at <24 weeks [14]. Royal College of Obstetricians and Gynecologists recommends AST to women between 24 + 0 and 34 + 6 weeks of gestation who are at risk of preterm birth. AST should be considered for women between 23 + 0 and 23 + 6 weeks of gestation who are at risk of preterm birth [15]. According to current guidelines of the Polish Gynecological Society, AST is recommended between weeks 24 and 36 of pregnancy in pregnant women at risk of a preterm delivery. The preferred drug is betamethasone, due to a larger number of available studies that confirm its effectiveness [16]. In the group studied by us, a lack of AST caused a twofold increase in the risk of IVH. To summarize the conclusions resulting from a variety of studies, the use of AST administered when indications exist is very important in reducing the risk of preterm-associated morbidities.

In our study group, we found that asphyxia represents one of the factors that increase the risk of severe IVH. Asphyxia is related to abnormal gas exchange which results in oxygen deficit and hypercapnea. In a study by Liu et al. [17] on 1122 infants born before week 37 of gestational age, asphyxia is mentioned as one of major risk factors for IVH. Severe asphyxia, defined as Apgar score of ≤3 at 1 and 5 min, is also listed as an IVH risk factor in infants with birth weight <1500 g (Adegoke et al.) [18]. These results from coagulation cascade disruptions caused by asphyxia, and from impaired aggregation of platelets. Even though some studies indicate that asphyxia per se does not cause neurodevelopmental impairment, hypoxia—which is its consequence—may result in serious morbidities. Even a small change in oxygen supply to the brain increases the risk of hemorrhage in the central nervous system. Even with appropriate saturation, cerebral regional oxygen saturation may be impaired, which considerably increased the risk of IVH in the group of preterm infants analyzed by Baik et al. [19]. In view of the above, prevention against and early diagnosis of asphyxia turns out to be important. Again, one should remember about AST that prevents RDS one of major causes of asphyxia.

In our study, the cut-off point for acidosis was set at pH below 7.2 and/or BE less than −10. For therapy sodium hydrogen carbonate (NaHCO_3_) was used. As shown in our analysis, acidosis increased the risk of severe IVH (grade 3 and 4) in preterm neonates born before 32 weeks. With regard to the intrinsic adverse effect of acidosis on the development of IVH, many studies take a consistent standpoint. A study by Randolph et al. [20] on a group of 3979 patients with VLBW, focusing on the effects of acidosis in preterm infants, indicated an increased risk of severe IVH as well as higher mortality rates and higher incidence rates of neurodevelopmental impairment. The study by Rong et al. [7] also listed acidosis among risk factors that have a disruptive impact on hemodynamic processes and thus play a role in the pathogenesis of IVH. In a study carried out by Adegoke et al. [18] on the group of 87 preterm infants with birth weight <1500 g the association of acidosis and IVH was also confirmed. Negative effects of acidosis are a result of its impact on hemostasis. Excessive acidity inhibits platelet aggregation, thus promoting hemorrhage. There is still discussion concerning therapy for acidosis. Administration of NaHCO_3_ is a commonly used method but its impact on the development of IVH is not obvious, as the results of studies are ambivalent. Xu et al. [21] investigated a group of 59 preterm neonates and concluded that the infusion of NaHCO_3_ directly changed the volume of blood in circulation and, indirectly, had an impact on the hemodynamics of cerebral circulation and, consequently, increased the risk of IVH. In a retrospective analysis of 3806 cases of children born <33 weeks of pregnancy Synnes et al. [22] demonstrated an association of the therapeutic use of NaHCO_3_ with IVH occurrence. They emphasize in their study that it is one of the modifiable risk factors and finding a better therapy might cause a decrease in that risk. Other sources, on the other hand, do not confirm the adverse impact of NaHCO_3_. A study by Beveridge and Wilkinson [23] demonstrated that there were no differences between sodium hydrogen carbonate infusion versus dextrose with regard to IVH, encephalopathy, and neonatal seizures. Even though no risk increase related to the use of NaHCO_3_ was detected, the authors noted that there was insufficient research evidence to determine whether the therapy actually reduced mortality in infants. While it is important to effectively prevent and treat acidosis, there is insufficient evidence to justify an intervention using NaHCO_3_.

Another IVH risk factor which was detected in our study was the delivery that took place at a facility other than a tertiary referral center. Tertiary referral centers are public academic and research hospitals. They carry out academic research, have more specialized equipment and highly trained staff. These are the likely reasons why delivery at a hospital other than a tertiary referral center was related to a higher risk of IVH. Another factor contributing to an increased risk might have been the transport of premature neonates from lower-level referral centers to our hospital. As shown in an American study comprising 67,596 infants with birth weight <1500 g born between 1997 and 2004, moving the infant from one hospital to another increased both the frequency and the severity of IVH. It is not completely clear what contributed to the increase in risk, but the aspects considered were vigorous manipulations, kinking or obstruction of the endotracheal tube, self extubation, or iatrogenic trauma during neonatal transport [24]. Similarly, Chinese research also confirmed the adverse effect of neonatal transport on the development of IVH [7]. In addition to the aspects listed above, variations in ambient temperature were also considered to be a likely cause. With the minimization of potential risk in mind, women at risk of premature delivery and perinatal complications should, whenever possible, give birth at facilities with higher reference status.

## Conclusions

No or uncompleted antenatal steroid therapy increased probability for development of severe intraventricular hemorrhage. Antenatal steroids therapy should be promoted among women at risk of a premature delivery. Hypotension therapy with catecholamines and acidosis with sodium hydrogen carbonate should be carefully considered. The use of appropriate prophylaxis of perinatal (antenatal steroids therapy women at risk of preterm birth, limiting the indications for the use of catecholamines for hypotension treatment and sodium hydrogen carbonate for acidosis therapy, limitation of preterm deliveries outside tertiary referral centeres) significantly reduces the incidence of intraventricular hemorrhage stage 3 and 4. The significance of intraventricular hemorrhage creates a need to carry out periodical analysis, at regional level, concerning its incidence, causes and effects to improve local treatment outcomes by identifying further courses of action.
